# Conjugation to a cell-penetrating peptide drives the tumour accumulation of the GLP1R antagonist exendin(9-39)

**DOI:** 10.1007/s00259-022-06041-y

**Published:** 2022-11-30

**Authors:** Estel Collado Camps, Sanne A. M. van Lith, Annemarie Kip, Cathelijne Frielink, Lieke Joosten, Roland Brock, Martin Gotthardt

**Affiliations:** 1 Department of Medical Imaging, Radboudumc, P.O. Box 9101, 6500 HB Nijmegen, The Netherlands; 2 Department of Biochemistry, Radboud Institute for Molecular Life Sciences, Radboudumc, Nijmegen, The Netherlands; 3 Present Address: Department of Tumour Immunology, Radboud Institute for Molecular Life Sciences, Radboudumc, 278 Tumor Immunology, Radboudumc, P.O. Box 9101, 6500 HB Nijmegen, The Netherlands; 4 Department of Medical Biochemistry, College of Medicine and Medical Sciences, Arabian Gulf University, Manama, Kingdom of Bahrain

**Keywords:** Exendin, Antagonist, Tracer, Cell-penetrating peptide, Cellular internalisation

## Abstract

**Purpose:**

Exendin, an analogue of the glucagon-like peptide 1 (GLP1), is an excellent tracer for molecular imaging of pancreatic beta cells and beta cell-derived tumours. The commonly used form, exendin-4, activates the GLP1 receptor and causes internalisation of the peptide-receptor complex. As a consequence, injection of exendin-4 can lead to adverse effects such as nausea, vomiting and hypoglycaemia and thus requires close monitoring during application. By comparison, the antagonist exendin(9-39) does not activate the receptor, but its lack of internalisation has precluded its use as a tracer. Improving the cellular uptake of exendin(9-39) could turn it into a useful alternative tracer with less side-effects than exendin-4.

**Methods:**

We conjugated exendin-4 and exendin(9-39) to the well-known cell-penetrating peptide (CPP) penetratin. We evaluated cell binding and internalisation of the radiolabelled peptides *in vitro* and their biodistribution *in vivo*.

**Results:**

Exendin-4 showed internalisation irrespective of the presence of the CPP, whereas for exendin(9-39) only the penetratin conjugate internalised. Conjugation to the CPP also enhanced the *in vivo* tumour uptake and retention of exendin(9-39).

**Conclusion:**

We demonstrate that penetratin robustly improves internalisation and tumour retention of exendin(9-39), opening new avenues for antagonist-based *in vivo* imaging of GLP1R.

**Supplementary Information:**

The online version contains supplementary material available at 10.1007/s00259-022-06041-y.

## Introduction

Exendin is a functional mimetic of glucagon-like peptide 1 (GLP1), an incretin produced by L-cells in the small intestine which binds to the GLP1 receptor (GLP1R). GLP1R is expressed on pancreatic beta cells, where it stimulates glucose-dependent insulin release. The higher *in vivo* stability and receptor affinity of exendin compared to GLP1 makes it an excellent GLP1 analogue for the treatment of type 2 diabetes. Currently, several exendin formulations are clinically used to help normalise glucose levels in people with type 2 diabetes [1, 2].

Furthermore, radiolabelled exendin is a valuable tracer for molecular imaging of GLP1R. Clinical studies show that exendin-based nuclear imaging using single-photon emission computed tomography (SPECT) or positron emission tomography (PET) is more sensitive than ultrasound, computed tomography (CT) or magnetic resonance imaging (MRI) alone for the detection of GLP1R-overexpressing insulinomas and of focal lesions in congenital hyperinsulinism [3–5]. Since uptake of radiolabelled exendin correlates with beta-cell mass in preclinical models [6], this tracer also holds great potential to study and understand the pathophysiology of diabetes [7].

The variant exendin(9-39) lacks the N-terminal part needed for receptor activation and internalisation, therefore being a GLP1R antagonist [8]. Although their affinities for GLP1R are similar [9], exendin-4 leads to rapid receptor activation and internalisation, while exendin(9-39) does not. Lack of internalisation of the antagonist is likely the cause of its negligible tumour accumulation, reported previously by our group for [^111^In]In-DTPA-exendin(9-39) in subcutaneous INS-1 tumours [9]. Only in *in vivo* studies with tumours highly overexpressing GLP1R as well as at very early timepoints after injection, exendin(9-39) showed good tumour accumulation. Otherwise, in models better resembling the clinical situation, exendin(9-39) accumulates only poorly [10–12].

An important advantage of antagonistic tracers is their lack of receptor activation, which prevents side effects. In the case of exendin-4, GLP1R stimulation causes adverse effects such as nausea, vomiting, hypoglycaemia and (mild) tachycardia [3, 4]. Currently, for safety reasons, monitoring and glucose infusion are applied during exendin-based imaging, especially to avoid severe hypoglycaemia [4]. This concern creates a strong rationale for the development of efficient antagonistic tracers. We hypothesised that increasing the cellular uptake of exendin(9-39) would increase tumour retention. This could open the way to using exendin(9-39) as a safe alternative tracer for GLP1R imaging.

Cell-penetrating peptides (CPPs) can trigger cellular uptake irrespective of receptor activation. CPPs are peptides with a length of 5 to 30 amino acids which are readily internalised by cells, in a cell-type and receptor-independent manner. The first CPPs were discovered in the 90s and are fragments of naturally occurring proteins [13, 14]. Penetratin (Pen) is one of them, being derived from the antennapedia homeobox protein of *Drosophila melanogaster*. Pen has been shown to drive the cellular internalisation of oligonucleotides [15, 16], anticancer drugs [17], peptides [18], and proteins [19, 20]. Importantly, we had previously established that conjugation of various CPPs to a nanobody binding the epidermal growth factor receptor (EGFR) increased endocytosis of the compound without increasing EGFR activation. This creates the rationale for combining penetratin and exendin variants for the GLP1R-directed approach [21, 22].

In the present study we compared exendin-4, exendin(9-39) and their respective C-terminal penetratin conjugates, exendin-4-Pen and exendin(9-39)-Pen, with regard to their GLP1R-dependent binding and internalisation *in vitro* and biodistribution and tumour retention *in vivo*.

## Methods

### Cell culture

The rat insulinoma cell line INS-1 [23] was cultured in RPMI-1640 medium supplemented with 10% foetal calf serum (FCS, PAN Biotech, Aidenbach, Germany), 2 mM glutamine, 10 mM HEPES, 50 μM β-mercaptoethanol and 1 mM sodium pyruvate, in a humidified atmosphere containing 5% CO_2_ at 37 °C.

The lung hamster cell line CHL was cultured in DMEM with 10% FCS, 4.5 mg/mL glucose, 2 mM glutamine, non-essential amino acids and 1 mM sodium pyruvate in a humidified atmosphere containing 5% CO_2_ at 37°C. For stably transfected GLP1R+ CHL cells, media contained 0.5 mg/mL geneticin (G-418 sulphate). CHL-GLP1R cells were a kind gift of Brigitte Lankat-Buttgereit, Philipps-Universität Marburg.

When not indicated otherwise, media and reagents were from Gibco, Life Technologies (Waltham, MA, USA).

### Peptide radiolabelling

Lys^40^DTPA-exendin-4, Lys^55^DTPA-exendin-4-Pen, Lys^31^DTPA-exendin(9-39) and Lys^46^DTPA-exendin(9-39)-Pen (Table 1) were purchased from Peptide Specialty Laboratories (Heidelberg, Germany).

**Table 1:** Sequences of the exendin variants used. The CPP (penetratin) is underlined, C. terminal additions to the original sequence are in bold. The chelator diethylenetriaminepentaacetic acid (DTPA) was attached to the side-chain of an extra lysin in the case of the unconjugated variants (exendin-4 and exendin(9-39)). For the conjugated variants (exendin-4-Pen and exendin(9-39)-Pen), the side chain of the last lysin present in the original sequence was used to attach the DTPA molecule

Peptide	Sequence
Exendin-4	HGEGTFTSDLSKQMEEEAVRLFIEWLKNGGPSSGAPPPS**K**-**DTPA**
Exendin(9-39)	DLSKQMEEEAVRLFIEWLKNGGPSSGAPPPS**K**-**DTPA**
Exendin-4-Pen	HGEGTFTSDLSKQMEEEAVRLFIEWLKNGGPSSGAPPPSRQIKIWFQNRRMKWKK-**DTPA**
Exendin(9-39)-Pen	DLSKQMEEEAVRLFIEWLKNGGPSSGAPPPSRQIKIWFQNRRMKWKK-**DTPA**

Peptides were labelled at a molar activity of 0.03 to 0.09 GBq/nmol (equivalent to a specific activity of 2.7 to 4.8 MBq/μg) for *in vivo* experiments, and with 0.04 to 0.07 GBq/nmol (equivalent to 4 MBq/μg) for *in vitro* experiments with ^111^InCl_3_ in 0.5 M 2-(N-morpholino)-ethanesulfonic (MES) buffer (metal free), pH 5.5, by incubation for 20 minutes at room temperature. After labelling, EDTA was added to a final concentration of 5 mM to chelate excess free indium. Tween-80 was added to a final concentration of 0.1% to reduce adsorption of the peptides to the surface of tubes and pipette tips. Labelling efficiency and radiochemical purity were determined by instant thin-layer chromatography (iTLC) on a silica gel chromatography strip (ITLC-SG, Agilent Technologies, Lake Forest, CA, USA), using 0.1 M EDTA in 0.1 M NH_4_Ac buffer, pH 5.5 as the mobile phase.

Radiochemical purity was also assessed by reversed phase high-performance liquid chromatography (RP-HPLC) on a C18 column (Alltima; 4.6 mm × 25 cm; Grace, Breda, The Netherlands). Peptides were injected in 20-μL water. The column was eluted with a linear gradient starting of 0.1% TFA (trifluoroacetic acid) in acetonitrile (3% to 100% in 10 min with a flow rate of 1 mL/min). For in vivo experiments, peptides were diluted in PBS with 0.5% BSA.

### Binding and internalisation assays

CHL and CHL-GLP1R cells were seeded into 6-well plates one or two days before the experiment and were used at 80% confluence. Exendin variants were radiolabelled following the procedure described above. Cells were incubated with approximately 50 pM (total activity around 1000 Bq) of the peptides in 1 mL RPMI-1640 containing 0.5% BSA (binding buffer) for 30 min or 4 h at 37°C. GLP1R binding specificity was assessed by co-incubation with 50 μg unlabelled exendin per well. After incubation, cells were washed twice with PBS, and the receptor-bound peptides were retrieved by incubation with ice-cold 0.1 M acetic acid, 154 mM NaCl and pH 2.6 for 10 minutes on ice. After washing twice with PBS, cells containing the internalized peptides were collected with 1 mL 0.1 M NaOH. Activity in both fractions was counted in a γ-counter (2480 Wizard 3”, LKB/Wallace, Perkin-Elmer, Waltham, MA, USA). Specific binding and internalization were calculated as percentage of the total activity added, using solution standards. Each condition was performed in triplicate (three wells, treated and measured separately).

### Animals

Animal experiments complied with the Dutch and European regulations on animal experimentation and were performed after approval of the Animal Ethical Committee of the Radboud University Nijmegen (project number: RU-DEC-2015-0071). Female BALB/c nude mice, 6–8 weeks old (Charles River Laboratories, L’Arbresle, France), were housed at the local animal facility in groups of 6, in IVC blueline cages enriched with bedding material and one polycarbonate shelter per cage. Animals were given at least 1 week to acclimatise before starting the experiments and had *ad libitum* access to water and chow. After tracer injection, some mice were housed individually overnight to avoid fighting or cross-contamination through radioactive material excreted in bodily fluids. Otherwise, solitary caging was avoided.

### In vivo biodistribution

Mice were injected subcutaneously on the right flank with 0.2 mL of a cell suspension containing 15,000,000 INS-1 cells/mL in RPMI (3,000,000 cells/mouse). When the tumours were 2–5 mm in diameter, mice were randomly divided into groups and the experiment was performed.

All mice were intravenously injected with 20 pmol/mouse (0.37 MBq/mouse) of the corresponding radiolabelled exendin variant in approximately 200 μL PBS/0.5% BSA. 12 mice received [^111^In]In-DTPA-exendin-4 (referred to as exendin-4), 12 mice received [^111^In]In-DTPA-exendin-4-Pen (referred to as exendin-4-Pen), 11 mice received [^111^In]In-DTPA-exendin(9-39) (referred to as exendin(9-39) and 11 mice received [^111^In]In-DTPA-exendin(9-39)-Pen (referred to as exendin(9-39)-Pen). To determine non-specific uptake, an excess of unlabelled peptide was injected (blocking group). A 100-fold excess of unlabelled DTPA-exendin-4 or DTPA-exendin(9-39) was co-injected (5 or 6 mice per group). Blocking groups were only included at the 4 h timepoint, to reduce the number of mice used as a control. After 1 h and 4 h after injection, mice were euthanized by CO_2_/O_2_ asphyxiation.

Blood, tumours and relevant tissues (muscle, heart, lung, spleen, pancreas, kidney, liver, stomach, duodenum and colon) of all animals were dissected, weighed and measured in a gamma-counter (2480 Wizard 3”, LKB/Wallace, Perkin-Elmer, Waltham, MA, USA). The percentage injected dose per gram tissue (% ID/g) was determined for each tissue, based on the cpm measured for diluted injection mixtures (standards).

### Data analysis, statistics and graphical representation

Plate assay data were plotted in GraphPad Prism. Data from the *in vivo* study were imported from Excel into R Studio (version 3.6.3 or higher) for plotting and statistical analysis. The following packages were used: tidyverse, readxl and patchwork. Where possible, we indicated the variability between biological replicates with dots, following the example of the “superplots” proposed by Lord et al. [24].

For the statistical analysis, the data were log transformed to correct for differences in the intra-group spread and ensure a normal distribution. The difference in tumour uptake between the four compounds was evaluated separately for each timepoint, with a one-way ANOVA and Tukey’s post hoc test. We accepted a type I alpha error of 0.05. The number of animals per group was selected assuming an SD of 10% of the mean, to reach a statistical power of 80% (beta type error = 0.2).

## Results

### Labelling and purity of exendin variants

Peptides were generated by solid-phase peptide synthesis. We added penetratin at the C-terminus of exendin to avoid interference with the N-terminal part of exendin-4, which is directly involved in receptor activation [25]. The chelator DTPA was coupled to an additional lysine at the C-terminus of the CPP.

[111In]In-exendin-4, [111In]In-exendin-4-Pen, [111In]In-exendin(9-39) and [111In]In-exendin(9-39)-Pen could be labelled with a high molar activity (0.04 to 0.07 GBq/nmol, equivalent to a specific activity of 4 MBq/μg), reaching a radiochemical purity of > 99%. HPLC profiles showed single peaks at the expected elution times, confirming the purity of the radiolabelled exendin variants (supplementary figure 1).

### Binding and internalisation of exendin variants

**Fig. 1:**
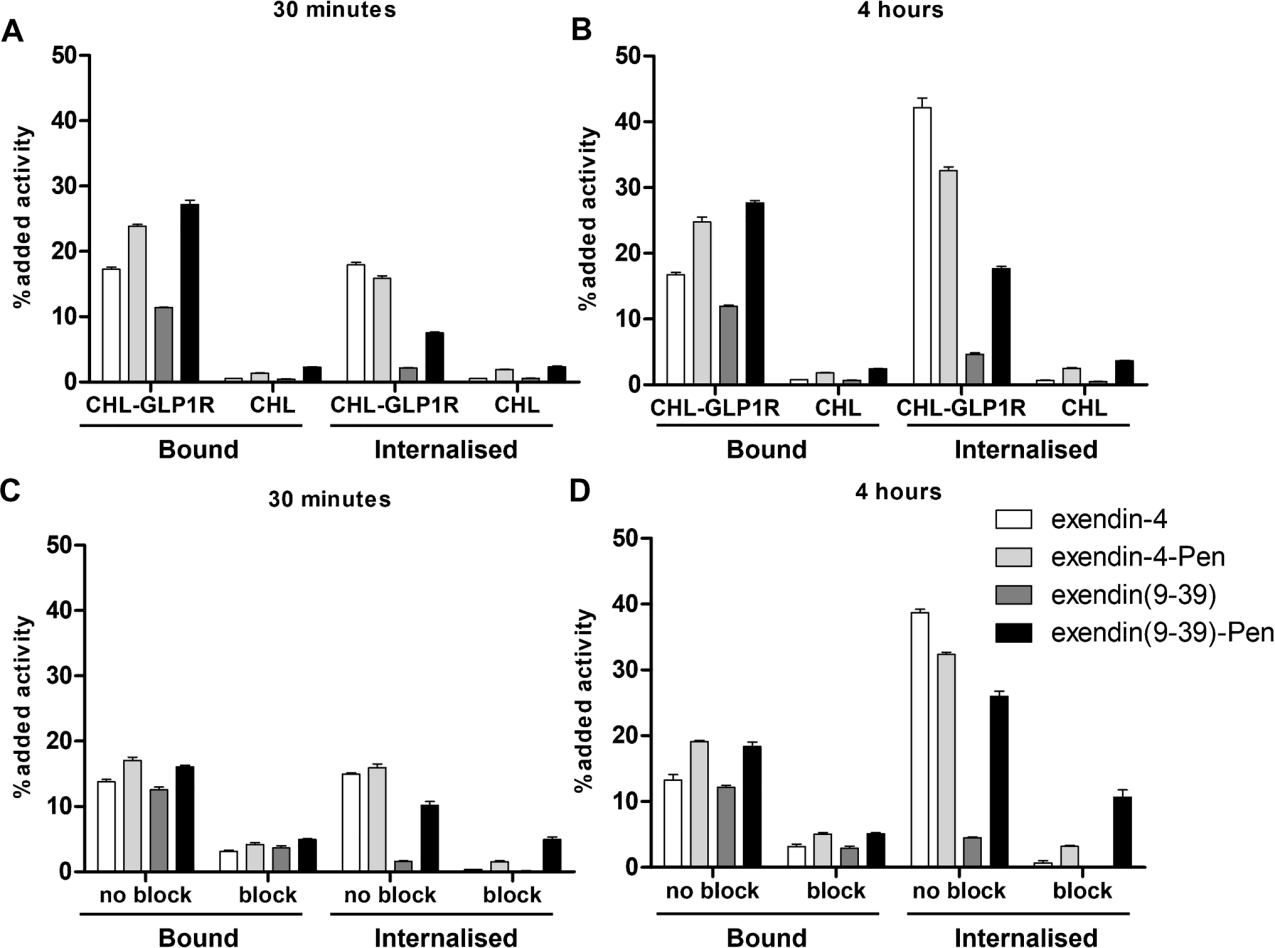
Binding and internalisation of exendin variants. Binding and internalisation on CHL-GLP1R and CHL cells after **A** 30 minutes incubation and **B** 4 hours incubation at 37 °C. Binding and internalisation on CHL-GLP1R cells, with and without an excess of unlabelled exendin-4 (block for exendin-4 and exendin-4-Pen) or exendin(9-39) (block for exendin(9-39) and exendin(9-39)-Pen), after **C** 30 minutes incubation and **D** after 4 hours incubation at 37 °C. Error bars indicate measurement SD within three independent wells, measured in the same experiment

First, we performed binding and internalisation assays with the radiolabelled exendin variants on CHL cells. To assess GLP1R specificity, we compared GLP1R-positive and GLP1R-negative cells (Fig. 1A, B). Additionally, an excess of unlabelled exendin-4 or exendin(9-39) was added to compete for binding with the radiolabelled compounds (Fig. 1C, D).

On GLP1R-positive cells, [111In]In-exendin(9-39)-Pen showed increased binding and internalisation compared to [111In]In-exendin(9-39) at 30 minutes and 4 hours incubation (internalisation being 7.5.2 ± 0.2% vs 2.2 ± 0.1% and 17.7 ± 0.5% vs 4.6 ± 0.4%, respectively; Fig. 1A, B). While internalisation increased over time, binding was similar at 30 minutes and 4 hours incubation for these compounds (Fig. 1). By comparison, [111In]In-exendin-4-Pen showed higher binding to CHL-GLP1R cells than [111In]In-exendin-4 after both 30 minutes and 4 hours incubation, but this did not translate in better internalisation.

All compounds showed low binding and internalisation in receptor negative CHL cells (Fig. 1A, B), and binding and internalisation decreased substantially when an excess of unlabelled exendin-4 or exendin(9-39) was added to receptor positive cells (Fig. 1C, D).

The generally lower binding in the assay shown in Fig. 1C, D than in the assay shown in Fig. 1A, B can probably be attributed to inter-experiment variability, as for each experiment the percentage of uptake is calculated in respect to the exact amount of activity added to the wells and there is some variation in both the amount of seeded cells and the added activity between assays. The relative differences between conditions were the same in both experiments (Fig. 1).

### In vivo biodistribution

To test whether the higher internalisation of exendin(9-39)-Pen compared to exendin(9-39) would enhance receptor-specific tumour accumulation *in vivo*, we performed a biodistribution study. Nude BALB/c mice with a subcutaneous INS-1 tumour were intravenously injected with the corresponding radiolabelled peptide. While CHL cells and their GLP1R-positive counterparts (used above) are reliable and easy to handle cell lines for in vitro testing of receptor specificity, we considered INS-1 cells preferable for *in vivo* studies because our group previously demonstrated that results obtained with INS-1 xenografts are more representative of the physiological situation [23]. Organs were collected 1 hour and 4 hours post-injection. A full overview of both timepoints for all four compounds can be found in supplementary figure 2.

**Fig. 2:**
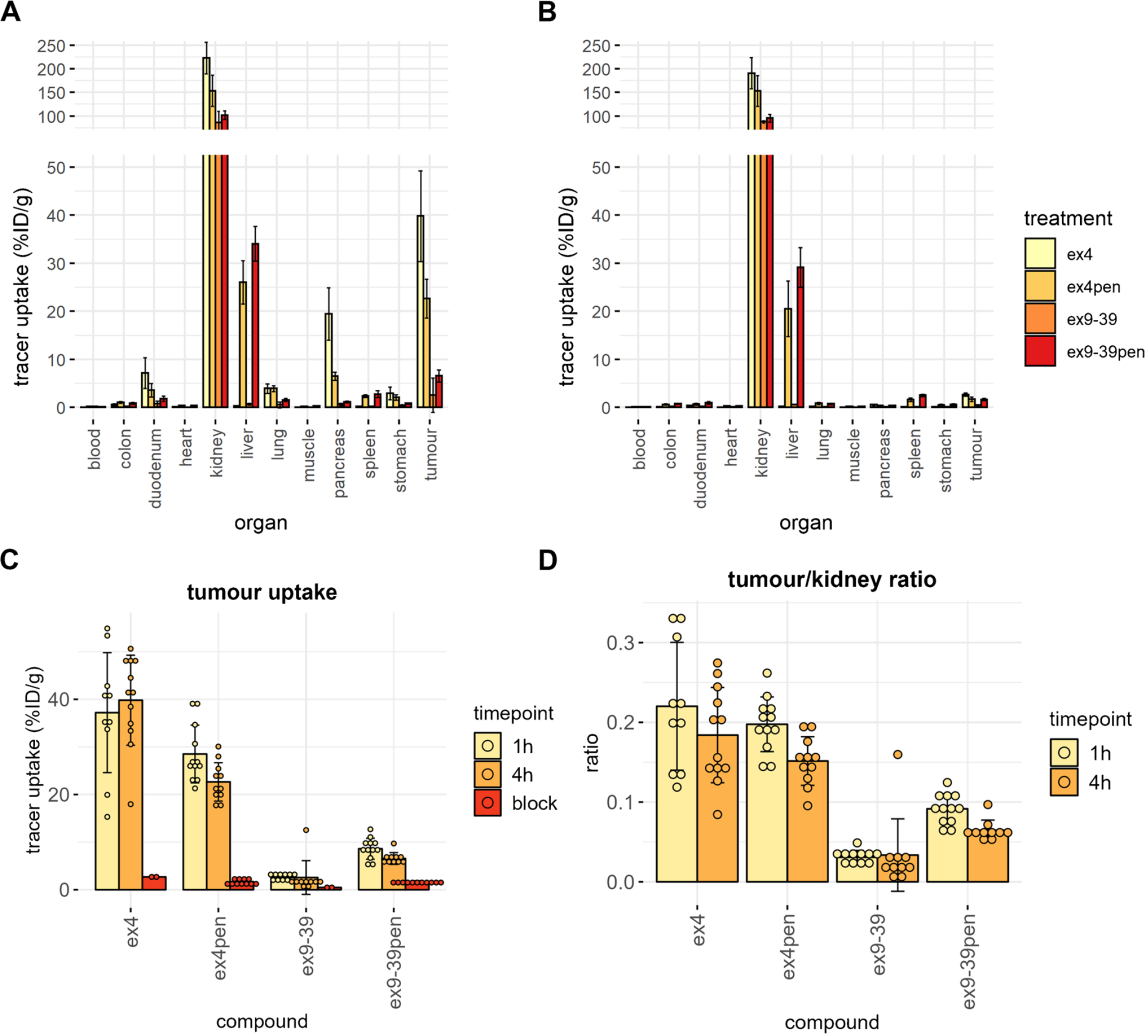
In vivo biodistribution of the four exendin variants after 4 h **A** without and **B** with block. BALB/c nude mice with subcutaneous INS-1 tumours were injected with 20 pmol of the corresponding compound. For the blocking condition, a 100x excess of either unlabelled exendin-4 (for exendin-4 and exendin-4-Pen groups) or unlabelled exendin(9-39) (for exendin(9-39) and exendin (9-39)-Pen groups) was administered together with the labelled compound. **C** Tumour uptake of all conjugates. Differences in tumour uptake were tested separately for each timepoint (1 h and 4 h) with a one-way ANOVA with post hoc Tukey’s correction. At 1 h, all groups significantly differed from each other in tumour uptake (*p* < 0.000001), except exendin-4 vs. exendin-4-Pen (*p* = 0.3). The same holds for the 4 h timepoint, where *p* < 0.0001 for all comparisons except for exendin-4 vs. exendin-4-Pen (*p* = 0.07). **D** Tumour/kidney ratios

The two antagonist variants showed significantly less tumour uptake than the two agonist variants, at both timepoints (Fig. 2A, B, supplementary figure 2). However, [111In]In-exendin(9-39)-Pen showed higher tumour accumulation (8.6 ± 2.2 %ID/g after 1 h and 6.6 ± 1.2 %ID/g after 4 h) compared to unconjugated [111In]In-exendin(9-39) (2.7 ± 0.7 %ID/g after 1 h and 2.6 ± 3.5 %ID/g after 4 h) (Fig. 2C). [111In]In-exendin(9-39)-Pen also showed higher tumour-to-kidney ratios compared to [111In]In-exendin(9-39) (0.09 ± 0.02% vs. 0.03 ± 0.01% after 1 h and 0.06 ± 0.01% vs. 0.03 ± 0.05% after 4 h, note that the high SD is due to an outlier) (Fig. 2D). In contrast, [111In]In-exendin-4-Pen showed lower tumour accumulation (28.5 ± 6 %ID/g after 1 h and 22.7 ± 4 %ID/g after 4 h) than unconjugated [111In]In-exendin-4 (37.2 ± 12.6 %ID/g after 1 h and 39.8 ± 9.4 %ID/g after 4 h), although this difference was non-significant (Fig. 2C). Tumour accumulation was specific for all four compounds, as it was blocked by an excess of non-labelled ligand (Fig. 2C).

Pancreatic uptake was high for [111In]In-exendin-4 (17.2 ± 4.3 %ID/g after 1 h and 19.5 ± 6.5 %ID/g after 4 h), and lower for [111In]In-exendin-4-Pen (8.8 ± 1.7 %ID/g after 1 h and 6.5 ± 0.8 %ID/g after 4 h). For the antagonist, addition of penetratin had no effect on pancreatic uptake. Both antagonistic variants showed very low uptake (1.7 ± 0.5 %ID/g after 1 h and 2.2 ± 0.3 %ID/g after 4 h for [111In]In-exendin(9-39) and 2.2 ± 0.3 %ID/g after 1 h and 1.2 ± 0.2 %ID/g after 4 h for [111In]In-exendin(9-39)-Pen) (Fig. 2A, B).

Both [111In]In-exendin-4-Pen and [111In]In-exendin(9-39)-Pen showed unspecific accumulation in the liver and a small increase in spleen accumulation in comparison to the unconjugated peptides as well.

## Discussion

The objective of this study was to investigate the effect of the CPP penetratin on the cell and tissue interactions of exendin-4 and its antagonistic analogue exendin(9-39). We were especially interested in the opportunity to improve exendin(9-39) retention in GLP1R-expressing tissues. We found that penetratin increases binding and internalisation of exendin(9-39) *in vitro* and specific tumour uptake *in vivo*. Our results provide proof of concept that CPP conjugation can be used to turn a non-internalising antagonist into an internalizing tracer molecule for molecular imaging and theranostics.

*In vitro*, penetratin led to increased binding and uptake of [111In]In-exendin(9-39) in GLP1R-expressing cells. In contrast to binding, internalisation could not be completely blocked by an excess of unlabelled exendin(9-39). This observation is consistent with the presence of receptor unspecific internalisation triggered by the CPP. However, uptake of the exendin-CPP conjugates was low in cells that do not express GLP1R (Fig. 1A, B). This shows that efficient uptake only occurs when interaction of the ligand with the receptor is present. We have previously observed similar combined effects for penetratin and the nanobody 7D12 [22].

In contrast to what we found for exendin(9-39), conjugation of exendin-4 to penetratin did not lead to an increase in internalisation. A slight increase in binding was observed, which is probably due to interaction of the CPP with the cell membrane. Exendin-4 internalisation was very efficient, as has been reported before [9].

The observations we made for exendin(9-39)-Pen raise new questions for the CPP field. A current working hypothesis proposes that CPPs trigger internalisation by cross-linking of glycosaminoglycans [26]. Observations supporting this hypothesis were typically made by following the uptake of fluorescently labelled CPPs. Fluorescence-based assays require concentrations in the lower micromolar range, while the 50-pM concentration used in our radioactivity-based assay is about five orders of magnitude smaller. Extensive cross-linking of glycosaminoglycans seems unlikely at pM concentrations, even if exendin(9-39)-mediated receptor binding will certainly contribute to some enrichment of the peptide at the plasma membrane. To our knowledge, it is the first time that CPP internalisation is studied at such low concentrations, and the scope of the work we present does not allow speculation on possible mechanisms. We hope these observations will motivate new interdisciplinary research, complementing fluorescence-based assays with other approaches, to gain a deeper understanding of the capacity of CPPs to trigger endocytosis.

The results *in vivo* reflected the differences observed *in vitro* between agonist and antagonist conjugates. [111In]In-exendin(9-39)-Pen reached a higher tumour uptake in comparison to [111In]In-exendin(9-39) (Fig. 2C). Importantly, [111In]In-exendin(9-39)-Pen showed higher tumour-to-kidney ratios than unconjugated [111In]In-exendin(9-39) (Fig. 2D). In contrast, [111In]In-exendin-4-Pen did not show higher tumour accumulation than unconjugated [111In]In-exendin-4. Considering the *in vitro* data, increased tumour uptake of [111In]In-exendin(9-39)-Pen is likely due to internalization of the tracer and subsequent intracellular trapping of the residualizing complex [^111^In]In-DTPA. This confirms our hypothesis that CPP-mediated internalisation leads to higher tissue accumulation of the antagonist.

[111In]In-exendin-4 showed considerable uptake into the pancreas which, however, was lower for the [111In]In-exendin-4-Pen conjugate. This difference in uptake may be explained by sequestration of the CPP conjugate in the liver. In contrast, neither [111In]In-exendin(9-39) nor [111In]In-exendin(9-39)-Pen showed increased pancreatic uptake. However, one must be cautious in drawing conclusions from mouse pancreatic uptake. The mouse exocrine pancreas takes up exendin in a GLP1R unspecific manner, which does not reflect the human situation. Rats are a more suitable model for pancreatic uptake, as our group previously reported [27].

Both [111In]In-exendin-4-Pen and [111In]In-exendin(9-39)-Pen showed unspecific liver uptake, which was not observed for the exendin analogues without penetratin. For [111In]In-exendin-4, the increased hepatic sequestration of the penetratin conjugate correlated with a decreased distribution to the pancreas and tumours. By comparison, for the [111In]In-exendin(9-39)-Pen conjugate this was not the case. Liver sequestration is a common phenomenon among CPPs [26, 28], and we also observed it upon conjugation of penetratin to a nanobody. The positive charges of the CPPs are often named as a possible reason, but the mechanism has not been defined in detail. For tumour imaging, the only negative influence of high liver uptake could be in the detection of tumours close to the liver or of liver metastases. Otherwise, it would not pose serious health risks. However, decreasing liver uptake could lead to even higher tumour uptake. We showed for the nanobody 7D12 that CPPs with different physicochemical properties differentially affect the interaction of their conjugates with cells [22, 29]. A next challenge will be to identify CPP-ligand pairs that enhance accumulation at the target site with little hepatic accumulation. It would be interesting to assess if less charged CPPs, or shielded activatable CPPs [30–32] can achieve this goal. Furthermore, as the effects of N-terminal conjugation instead of C-terminal conjugation are conjugate dependent [33], it would be worthwhile to test N-terminal CPP conjugation of exendin(9-39). Finally, CPPs containing D-amino acids could be beneficial for uptake, as they have higher proteolytic stability [17, 34, 35].

A tracer with the characteristics of exendin(9-39)-Pen is likely to quickly find a way to application. Our group provided proof of concept that exendin can be used for image-guided surgery (IGS) and targeted photodynamic therapy (tPDT) [36, 37]. These techniques have great theranostic potential but require high pharmacological doses of the tracer, increasing the risk of side-effects when using an agonist. This complication underlines the need for effective antagonistic tracers to avoid side effects. Furthermore, as for tPDT and IGS fluorophores are locally activated by light application, tracer accumulated in other organs (e.g., the liver) remains inactivated and will thus neither disturb detection of tumour tissues nor cause off-target toxicity.

In this study, we restricted ourselves to a proof-of-concept of increased antagonist internalisation and increased tumour retention. When aiming at clinical applications, further work will have to address the potential toxicity of the conjugated antagonist. We consider this risk low as for free penetratin cellular toxicity is only observed at concentrations higher than 10 μM.

Importantly, this is the first study that investigated the impact of CPP conjugation for an antagonistic G-protein-coupled receptor peptide ligand from *in vitro* to *in vivo*. Previously, non-arginine was shown to increase the *in vitro* uptake of a peptide conjugate consisting of bombesin and an endosome-disrupting peptide, aiming at the cytosolic delivery of plasmid DNA [38]. However, for these conjugates, no *in vivo* data have been presented. The previous investigation that most resembled our approach was the N- and C-terminal conjugation of several CPPs to the agonistic peptide PTH(1-34), derived from the parathyroid hormone (PTH) [33]. The IC_50_ values and *in vitro* epithelial permeability were assessed, but not cellular internalisation or biodistribution. Interestingly, that study showed that C- and N-terminal conjugation of the CPP changed the properties of the PTH(1-34) conjugate, in a different way for each CPP [33]. This ties back to the point discussed above.

In conclusion, we showed for the first time that a CPP efficiently causes cellular internalisation of an antagonistic, non-internalising peptide ligand, thereby increasing tumour retention of the tracer *in vivo*. This result opens the door to further unleashing the great potential of exendin, as a research tool and as a theranostic agent. Future research into CPP conjugates should be extended to other non-internalising peptide antagonists.

## Supplementary Information

ESM 1 (DOCX 1404 kb)

## Abbreviations

CHL: Chinese hamster lung cell line
cpm: counts per minute
CPP: cell-penetrating peptide
DTPA: diethylenetriaminepentaacetic acid
EGFR: epidermal growth factor receptor
GLP1: glucagon-like peptide 1
GLP1R: glucagon-like peptide 1 receptor
INS-1: insulinoma 1 cell line
iTLC: thin-layer chromatography
%ID/g: percentage of injected dose per gram of tissue
IGS: image-guided surgery
MES: 2-(N-morpholino)ethanesulfonic acid
Pen: penetratin
PET: positron emission tomography
RP-HPLC: reverse-phase high-pressure liquid chromatography
SPECT: single photon emission computed tomography
tPDT: targeted photodynamic therapy
