# Supplementary material for: Conjugation to a cell-penetrating peptide drives the tumour accumulation of the GLP1R antagonist exendin(9-39)
Source: Eur J Nucl Med Mol Imaging. 2022 Nov 30;50(4):996–1004. doi: 10.1007/s00259-022-06041-y (PMC9931918; doi:10.1007/s00259-022-06041-y)
Supplement: Supplementary file 1 — (DOCX 1404 kb) [file 259_2022_6041_MOESM1_ESM.docx]

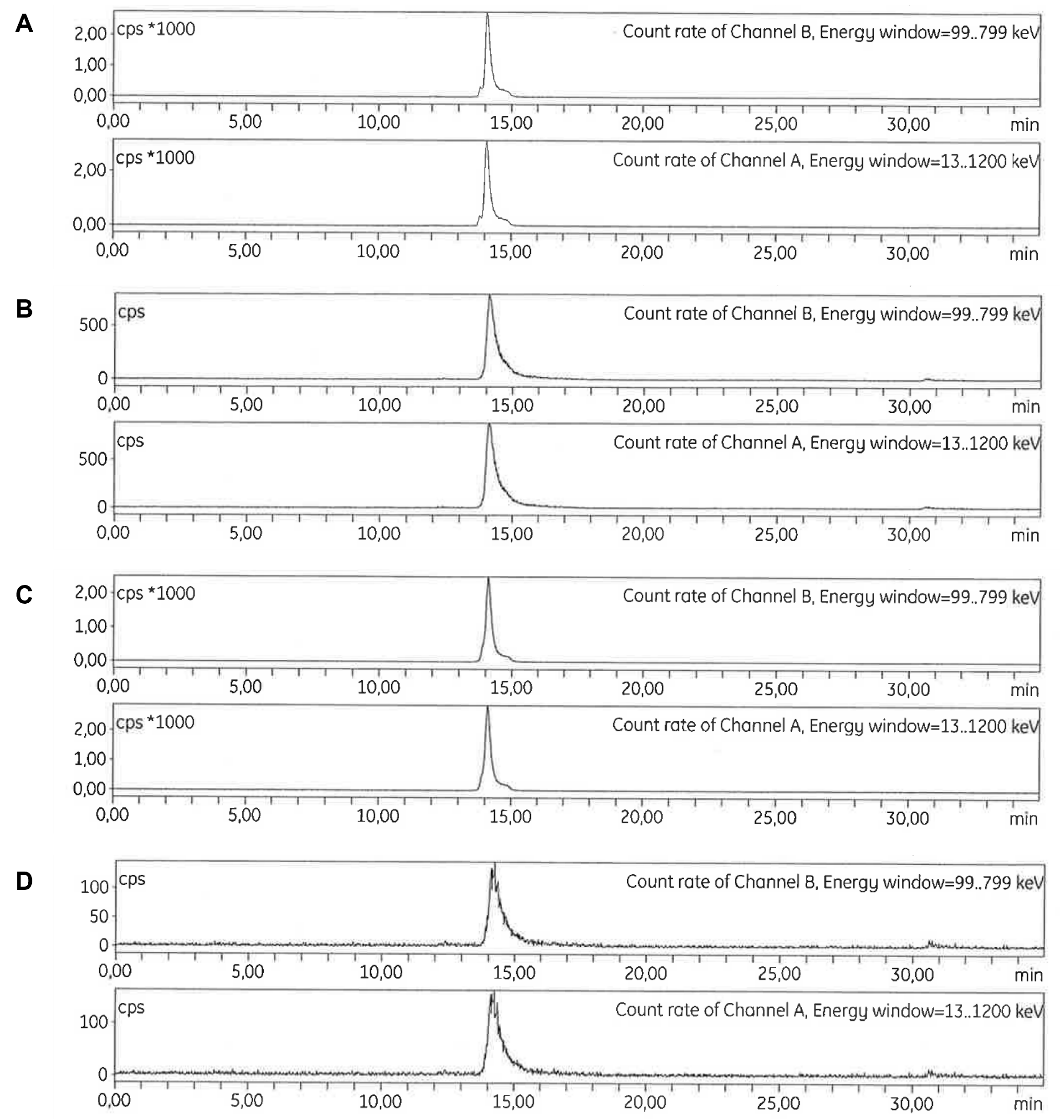


**SUPPLEMENTARY FIGURE 1.** All exendin analogues show uniform, radioactively labelled products. HPLC profile of radiolabelled exendin-4 (A), exendin-4-Pen (B), exendin(9-39) (C), and exendin(9-39)-Pen (D).


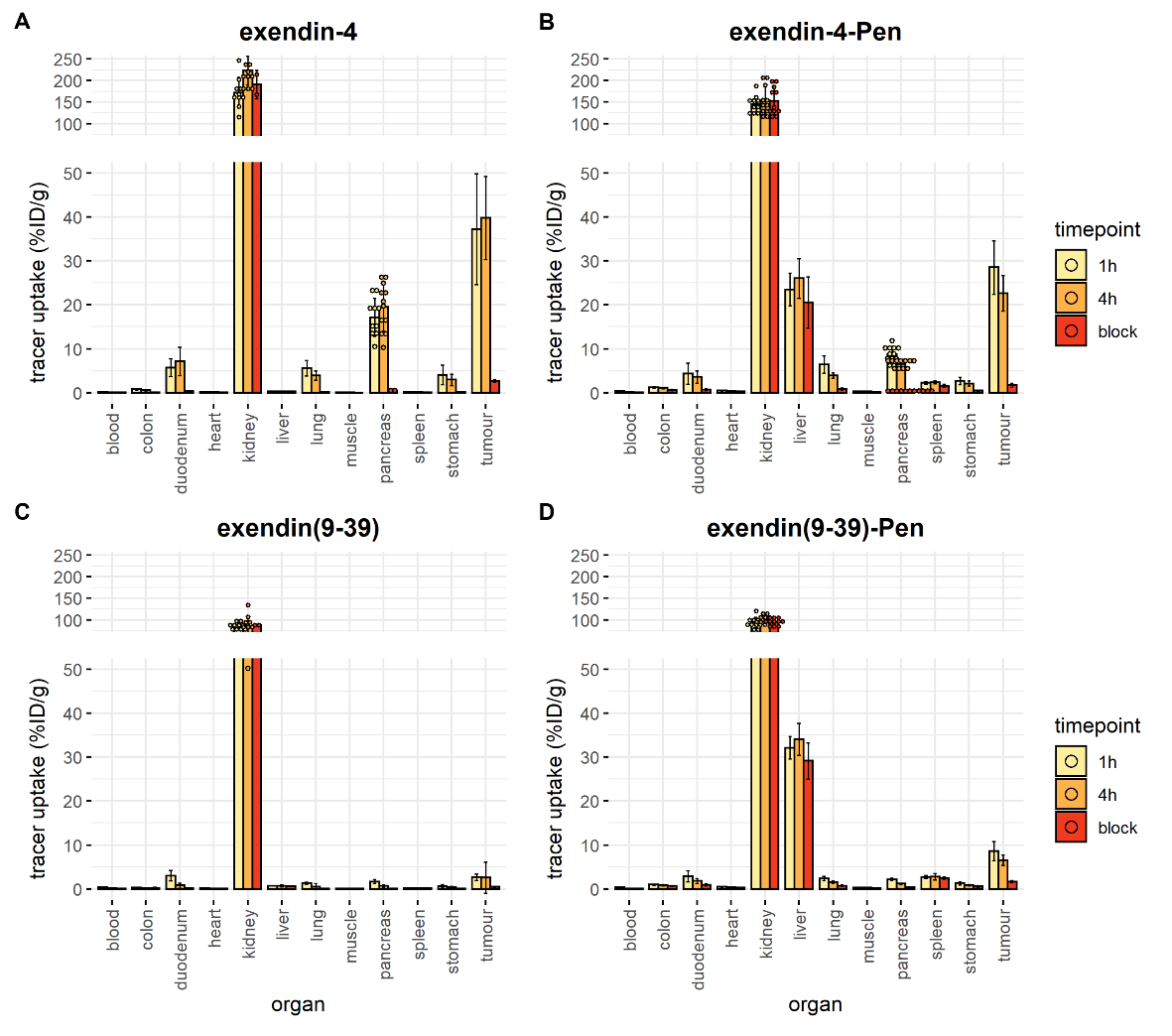


**SUPPLEMENTARY FIGURE 2.** In vivo biodistribution, overview per exendin variant. Balb/c nude mice with subcutaneous INS-1 tumours were injected with 20 pmol of the corresponding compound. For the blocking condition, a 100x excess of either unlabelled exendin-4 (for exendin-4 and exendin-4-Pen groups) or unlabelled exendin(9-39) (for exendin(9-39) and exendin (9-39)-Pen groups) was administered together with the labelled compound. Blocking condition was only measured at 4h. A) overview for exendin-4 B) overview for exendin-4-Pen C) overview for exendin(9-39) D) overview for exendin(9-39)-Pen.


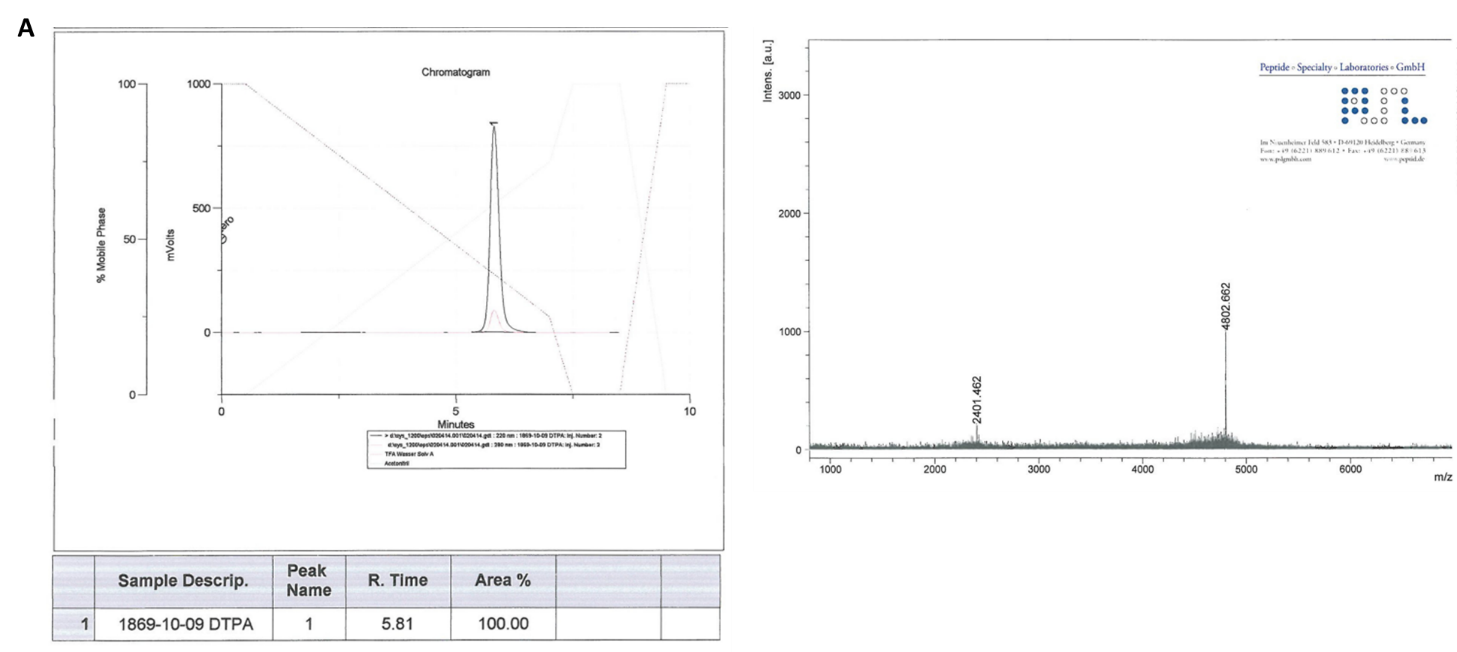


**
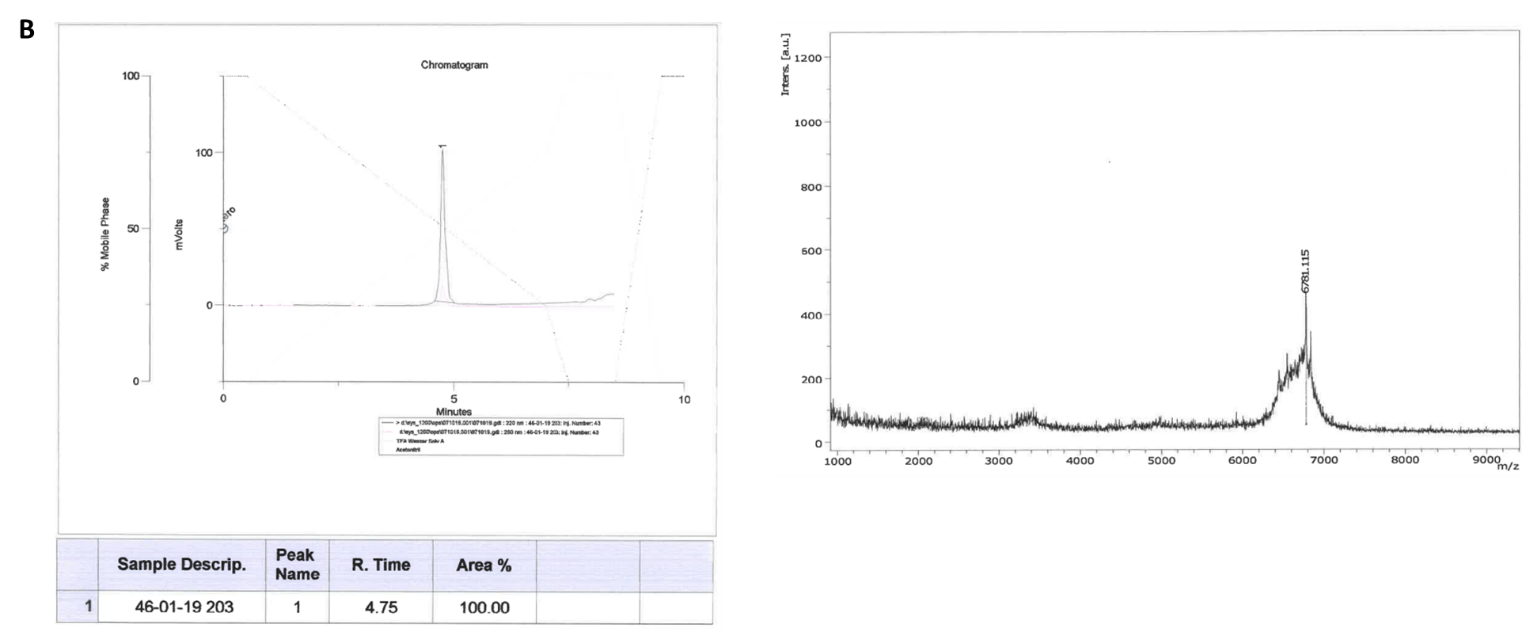
**

**
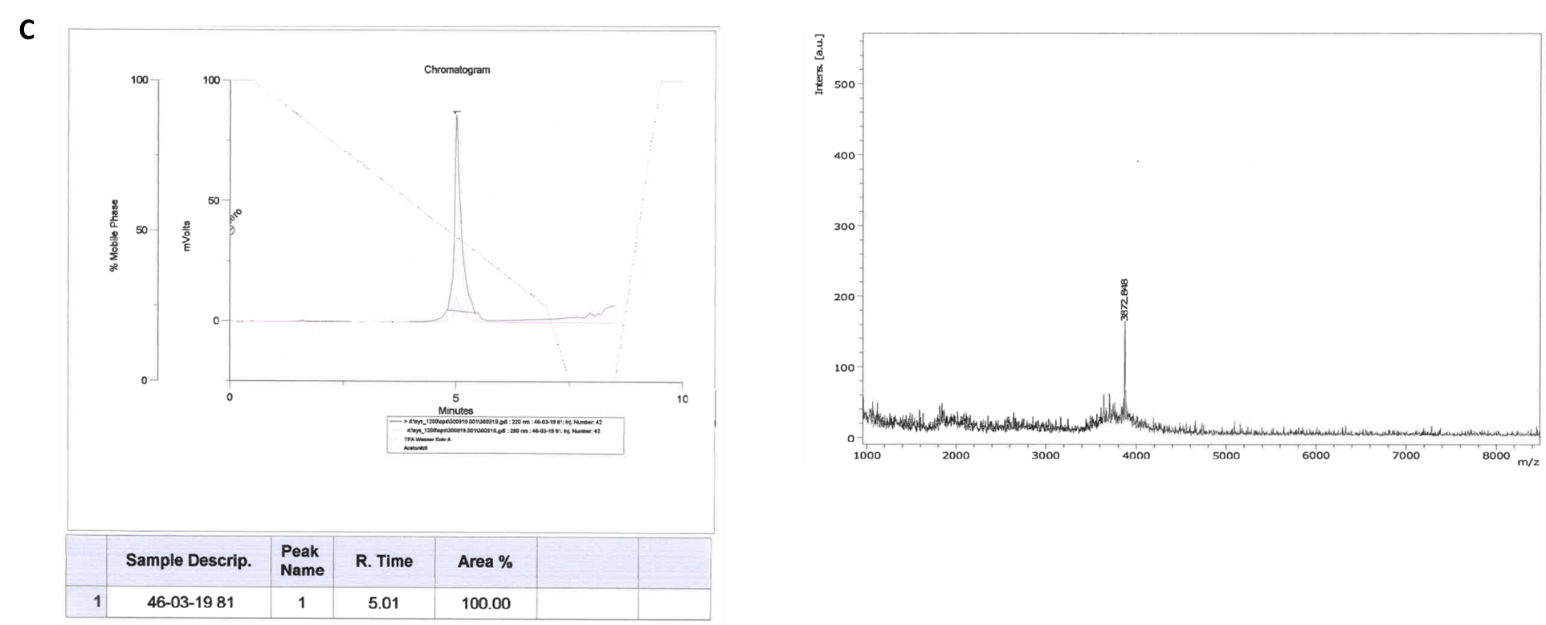
**

**
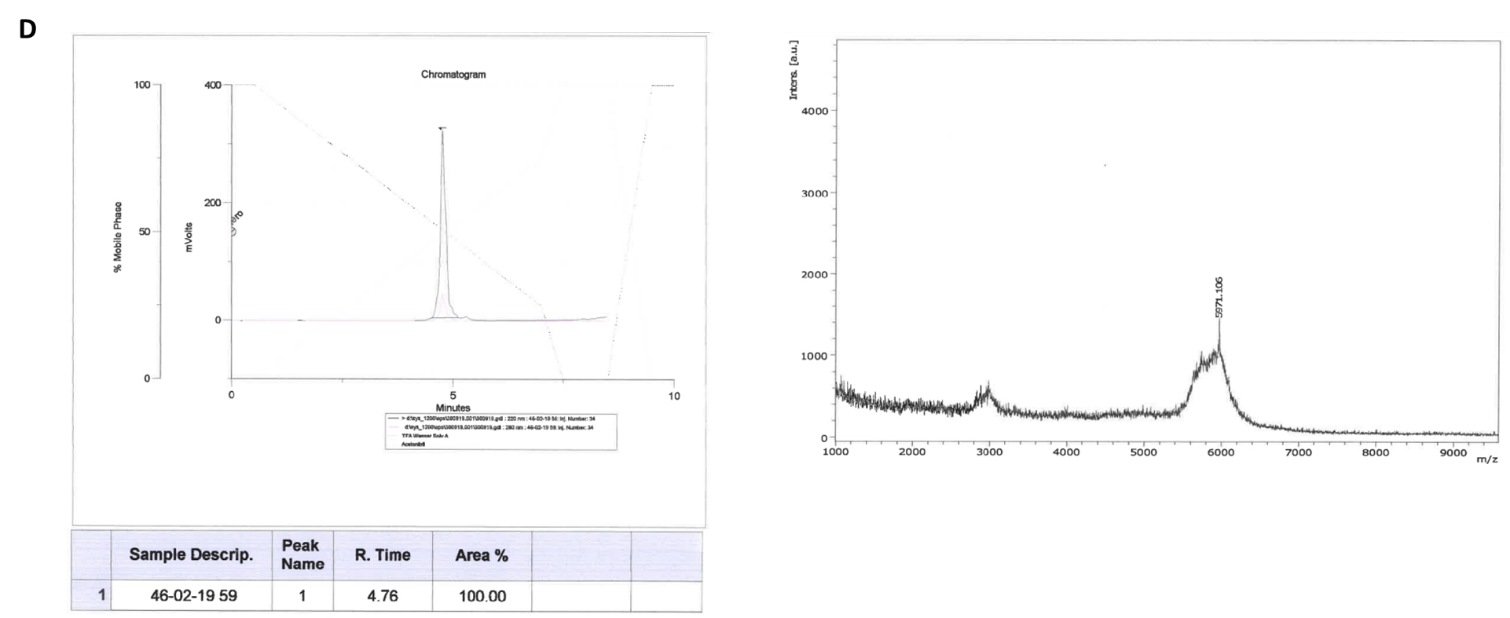
**


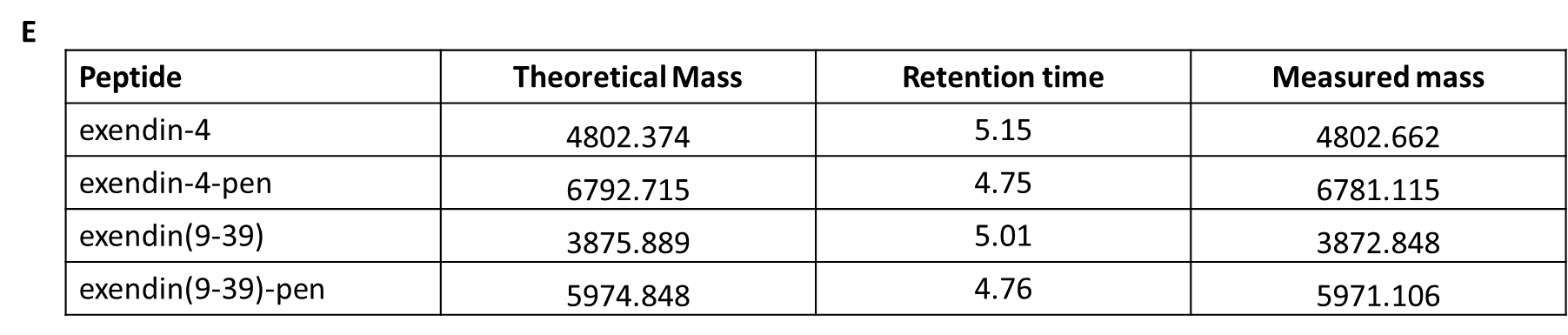


**SUPPLEMENTRAY FIGURE 3**. HPLC-MS profiles of A) exendin-4-DTPA, B) exendin-4-Pen-DTPA, C) exendin(9-39), D) exendin(9-39)-DTPA. E) masses and retention times of all four peptides (including a C-terminal DTPA moiety, which in the case of exendin-4-DTPA is attached to an Ahx linker)
